# Impacts of multiple anthropogenic stressors on stream macroinvertebrate community composition and functional diversity

**DOI:** 10.1002/ece3.6979

**Published:** 2020-12-16

**Authors:** Noel P. D. Juvigny‐Khenafou, Jeremy J. Piggott, David Atkinson, Yixin Zhang, Samuel J. Macaulay, Naicheng Wu, Christoph D. Matthaei

**Affiliations:** ^1^ Department of Evolution, Ecology and Behaviour University of Liverpool Liverpool UK; ^2^ Department of Health and Environmental Sciences Xi'an Jiaotong‐Liverpool University Jiangsu China; ^3^ iES – Institute for Environmental Sciences Landau University Koblenz‐Landau Landau Germany; ^4^ Trinity Centre for the Environment & Department of Zoology School of Natural Sciences Trinity College Dublin The University of Dublin Dublin Ireland; ^5^ Department of Landscape Architecture Gold Mantis School of Architecture Soochow University Suzhou China; ^6^ Department of Zoology University of Otago Dunedin New Zealand

**Keywords:** Alpha diversity, China, functional diversity, functional traits, macroinvertebrates, mesocosms, multiple stressors

## Abstract

Ensuring the provision of essential ecosystem services in systems affected by multiple stressors is a key challenge for theoretical and applied ecology. Trait‐based approaches have increasingly been used in multiple‐stressor research in freshwaters because they potentially provide a powerful method to explore the mechanisms underlying changes in populations and communities. Individual benthic macroinvertebrate traits associated with mobility, life history, morphology, and feeding habits are often used to determine how environmental drivers structure stream communities. However, to date multiple‐stressor research on stream invertebrates has focused more on taxonomic than on functional metrics. We conducted a fully crossed, 4‐factor experiment in 64 stream mesocosms fed by a pristine montane stream (21 days of colonization, 21 days of manipulations) and investigated the effects of nutrient enrichment, flow velocity reduction and sedimentation on invertebrate community, taxon, functional diversity and trait variables after 2 and 3 weeks of stressor exposure. 89% of the community structure metrics, 59% of the common taxa, 50% of functional diversity metrics, and 79% of functional traits responded to at least one stressor each. Deposited fine sediment and flow velocity reduction had the strongest impacts, affecting invertebrate abundances and diversity, and their effects translated into a reduction of functional redundancy. Stressor effects often varied between sampling occasions, further complicating the prediction of multiple‐stressor effects on communities. Overall, our study suggests that future research combining community, trait, and functional diversity assessments can improve our understanding of multiple‐stressor effects and their interactions in running waters.

## Graphical abstract

The figure is the conceptual framework of the experiment and expectations.

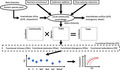

## 1 INTRODUCTION

Freshwater ecosystems worldwide are experiencing extreme anthropogenic pressures. Almost all river catchments are influenced directly (e.g., via point‐source pollution or physical changes) and/or indirectly (i.e., via global change) by human activities, with reducing freshwater biodiversity and hampering natural ecosystem functioning (Allan, 2004; Davis et al., 2010; Heathwaite, 2010). The large number of simultaneously or sequentially operating stressors renders multiple‐stressor studies a necessity in environmental research. However, such endeavors are not dominant in the literature, terminology and approaches variable across disciplines, and a unified framework to mechanistically understand the effects of multiple stressors is yet to be proposed (Côté et al., 2016; Nõges et al., 2016; Orr et al., 2020). Thus, lack of knowledge how stressors interact to shape ecological processes prevents stakeholders from making efficient short‐ and long‐term managerial decisions for conservation, restoration, or ecosystem services purposes (Lindenmayer et al., 2010).

Worldwide, stressors associated with land use drivers such as urban and agricultural development have become particularly pervasive (Dudgeon, 2019). They have accelerated biodiversity loss and ecosystem functioning declines via changes in the physicochemical parameters of streams, such as nutrient concentrations and their stoichiometric ratios, levels of deposited and suspended fine sediment, flow velocity and water turbidity (Gordon et al., 2008; Horváth et al., 2019; Wu et al., 2019). Fine sediment reduces habitat heterogeneity by infilling the interstitial spaces in the stream bed, smothers the filtering and breathing apparatus of invertebrates and increases water turbidity (Piggott et al., 2015). Fine sediment also covers biofilm and has direct negative effects on microbial communities (Piggott et al., 2015; Salis et al., 2017). Nutrient enrichment, mainly N and P derived from fertilizers, often produces subsidy‐stress response gradients in invertebrate communities (Wagenhoff et al., 2012; Woodward et al., 2012). Finally, flow velocity reduction, often resulting from water abstraction for irrigation, water translocation or water storage by dams (Dudgeon et al., 2006), modifies the physical habitat and the diffusion of material and individuals (Calapez et al., 2018; Lange et al., 2018; Wu et al., 2019). One anticipated interaction between these stressors is that a reduction in flow velocity facilitates sediment deposition and, thereby, local accumulation of chemicals and nutrients while decreasing water re‐oxygenation levels (Calapez et al., 2018).

To assess the effects of stressors on ecological stream health, benthic macroinvertebrate communities are often used (Bonada et al., 2006; Piggott et al., 2015). Certain groups of invertebrates such as Ephemeroptera, Plecoptera, and Trichoptera are highly sensitive to changes in their physicochemical environment (Bonada et al., 2006). Invertebrates also connect isolated water bodies across space and time, by dispersing over land and providing an important food source to higher trophic levels in both aquatic and adjacent riparian habitats (Sato et al., 2016). Further, macroinvertebrates are important drivers of stream‐wide ecosystem processes, for example by influencing the decomposition rate of organic matter or participating in secondary production (Frainer et al., 2018; Huryn & Benstead, 2019).

One important concept in ecology is the “environmental filtering” theory, which states that environmental conditions select for tolerant species and certain traits (Poff, 1997). While taxonomical assessments have often been used to assess the effects of multiple stressors on communities, they are bound to the regional species pool which reduces their potential for generalization. To overcome this limitation, trait‐based assessments, which rely on the compilation of community specific trait databases to characterize community niche breadth, have recently been getting more momentum (Ding et al., 2017). Trait assessments highlight the functional significance of species, that is, what they can do. Such assessments focus on the filtering role that environmental factors have in shaping community characterizations and provide mechanistic insights into community assembly and processes related to ecosystem functioning (Poff, 1997; Statzner & Bêche, 2010; Wu et al., 2019). Trait assessment results are not bound by the identity of species nor their regional pool but rather reflect functions individual can perform, thus facilitating the upscaling of local findings to larger geographical and longer temporal scales. In freshwater macroinvertebrate studies, traits associated with morphological characteristics, mobility, lifecycle, respiration strategy, and feeding habits have been very informative and can be linked to stream‐wide processes (Cummins, 2016; Dolédec et al., 2011; Poff et al., 2006). For instance, body shape and breathing apparatus are often associated with flow velocity and water oxygenation levels, two important components of microbial activity regulating stream‐wide processes such as nutrient cycling (Calapez et al., 2018; Dolédec et al., 2011). The developmental pace of individuals influences their tolerance to stressors (Dolédec et al., 2011) and their mobility moderates the linkage between habitats and the recovery of communities following stressor application (Guzman et al., 2019; Li et al., 2020; Schäfer et al., 2017). Finally, feeding habits directly link to the metabolic and stoichiometric resources needed by the individuals and thus to the dominant productivity pathways operating in a given stream such as primary or secondary productivity (Cummins, 2016; Frainer et al., 2016).

**FIGURE 1:**
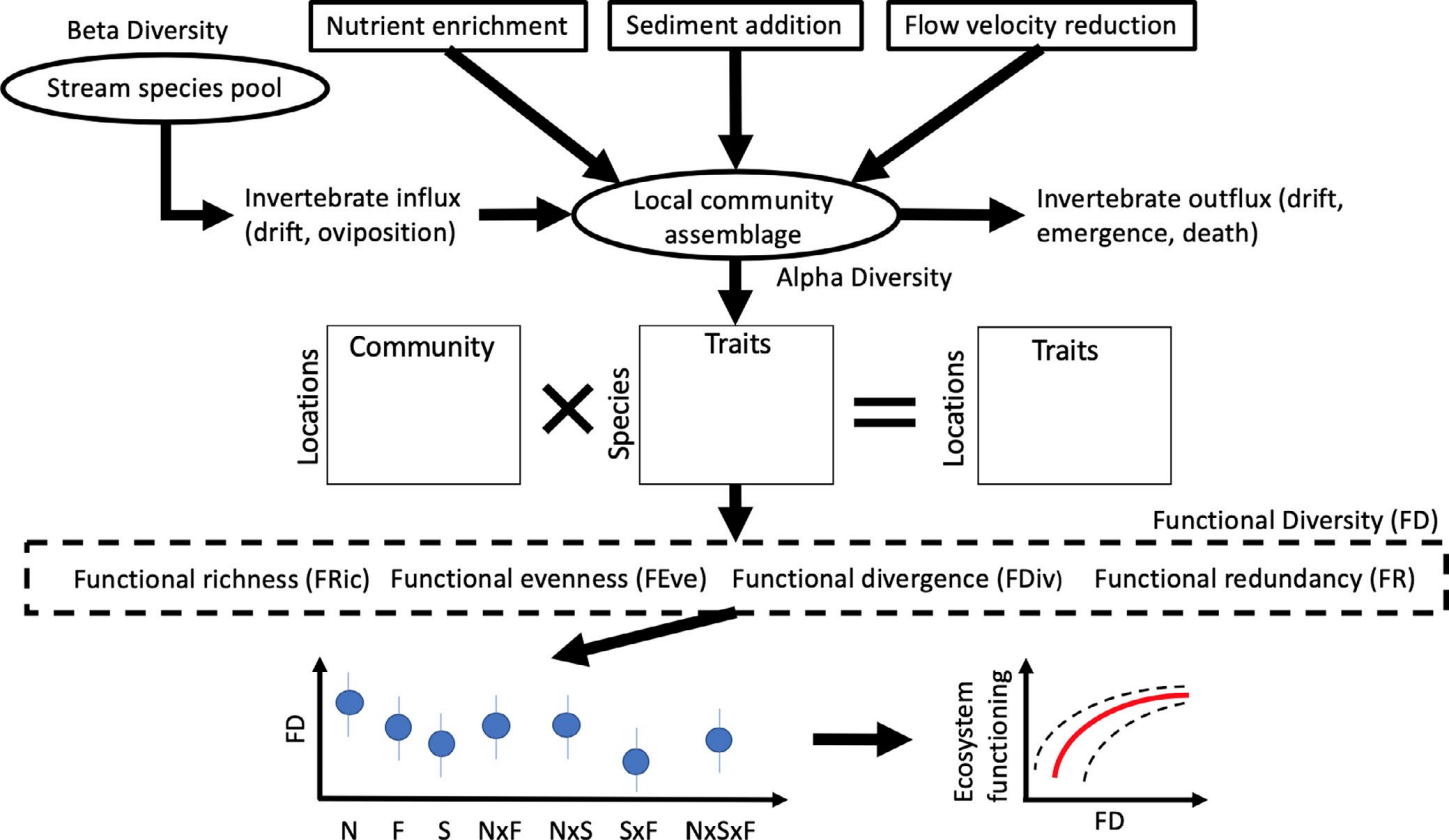
Conceptual model of the experiment. The benthic invertebrate community colonizing the stream mesocosms (stream community) is subject to different combinations of three stressors, nutrient enrichment (N), added fine sediment (S), and reduced flow velocity (F) at the mesocosm level. The resulting, filtered communities (local community) possess different densities of traits, which then influence functional diversity of the simulated stream ecosystem within each mesocosm. Functional diversity (FD) is defined by four metrics (FRic, FEve, FDiv, and FR). Nutrient enrichment is anticipated to promote diversity by increasing diversity and abundance of basal resources (e.g., periphyton), while sediment and flow velocity reduction have the opposite effect. We further anticipate antagonistic interactions between sediment addition and flow velocity reduction, whereas nutrient enrichment buffers the antagonistic effect of sediment and flow velocity reduction on functional diversity. Ecosystem functioning (not recorded in our study) is then expected to increase with functional diversity until reaching a plateau (Cardinale et al., 2012)

Despite linking macroinvertebrate communities to stream‐wide ecosystem functions such as decomposition and productivity, trait‐based assessments in multiple‐stressor research tend to be restricted to observational rather than manipulative field experiments (Ding et al., 2017; Dolédec et al., 2011; Mor et al., 2019). Further, mesocosm studies are an invaluable tool to ecologists by giving the ability to control and replicate multiple‐stressor treatments (Woodward et al., 2010). Therefore, more data from multiple‐stressor experiments conducted at the community and ecosystem levels in environmentally realistic scenarios are needed. To reduce this knowledge gap, we used field mesocosms to investigate the individual and combined effects of nutrient enrichment, flow velocity reduction and increased sedimentation on benthic stream macroinvertebrate communities and their associated functional traits (Figure 1). We aimed to determine whether macroinvertebrate communities are altered by stressor main effects and interactions through changes in functional trait diversity. Based on the findings of previous related research, we tested three specific hypotheses:

1. Sediment addition and flow velocity will have more pervasive stressor main effects than nutrient enrichment on community structure and trait composition because of their direct physical action on macroinvertebrates (Elbrecht et al., 2016);
2. Nutrient enrichment will enhance the biomass accumulation potential, either via an increase in the mesocosms’ carrying capacity or through a body‐size shift toward larger organisms, due to increased resource availability (Cross et al., 2015; Frost & Elser, 2002; Ott et al., 2014);
3. Interactions between flow velocity reduction and added sediment will be more common than interactions with nutrient enrichment (Matthaei et al., 2010);

## 2 MATERIALS AND METHODS

### 2.1 Experimental mesocosm system

The experiment was conducted in autumn from 1 October to 12 November 2018 on the bank of the Yinxi Stream outside the Jiulongfeng Nature Reserve in Huangshan, Anhui Province, China (30°07’07″N, 118°01′24″E, 330 m a.s.l.). Yinxi Stream is a near‐pristine, nutrient‐poor 2nd‐order montane stream (N‐NO_3_^−^ 0.39 ± 0.008 [mean ± *SE*] mg/L, N‐NH_4_^+^ 0.26 ± 0.002 mg/L, P‐PO_4_^+^ 0.01 ± 0.001 mg/L, pH 7.87 ± 0.018, conductivity 46.45 ± 0.029 µS/cm; four measurements each collected with a YSI Professional Plus, YSI Incorporated, at the mesocosm system's water intake point on 24 September 2018). The *ExStream* streamside mesocosm system comprised 64 circular flow‐through mesocosms, each with an external diameter of 25 cm and an inner outflow ring of 6 cm (volume 3.5 L; Microwave Ring Moulds, Interworld, New Zealand). More information about the experimental system and its location can be found in Juvigny‐Khenafou et al. (2020).

### 2.2 Experimental design

We manipulated fine sediment cover on the substratum surface, dissolved nutrient concentrations in the water column and flow velocity in 64 flow‐through stream mesocosms, with two nutrient treatments (ambient vs. enriched concentrations), two fine sediment treatments (ambient vs. added), and two flow velocities (fast vs. reduced; fast refers to our control) in a full‐factorial design. The experiment ran for 6 weeks with a 3‐week precolonization period (Day‐21 to Day‐1) followed by a 3‐week manipulation period (Day 0 to Day 21). Treatments were randomly allocated to 16 mesocosms within four blocks providing four replicates of each treatment combination across two sampling occasions (after 2 and 3 weeks of stressor exposure). Nutrient enrichment and flow velocity reduction started on Day 0 and both were continuously maintained throughout the manipulation phase. Also on Day 0, fine sediment was added in half of the mesocosms where it remained until Day 21.

Mesocosms were filled with 500 ml of dry substratum (>2 mm), ten 3–4 cm surface stones and one large stone (>6 cm) collected from the flood plain 200 m downstream of our experimental site. This substratum composition represented similar habitat heterogeneity as reported in other eastern catchments of the Yangtze River (Liu et al., 2016). Water current velocity in the mesocosms averaged 0.10 ± 0.008 m/s [*SE*] (recorded weekly in all channels using an OTT MF electromagnetic flow meter Pro; OTT HydroMet GmbH, Germany).

Flow began on 1 October 2018 (Day −21) to allow natural colonization of drifting stream organisms into the system. On Day‐4, macroinvertebrates were collected from riffles in the Yinxi Stream upstream of the pump intakes by kick‐netting for 3 min over an area of ~0.36 m^2^ (comparable to the benthic surface area of eight mesocosms). These invertebrates were added to the mesocosms to supplement natural colonization by taxa underrepresented in the drift (Elbrecht et al., 2016; Piggott et al., 2015). After collection, each kick‐net sample was subdivided into eight equal portions using a subsampler and then randomly distributed to individual mesocosms (one portion per mesocosm) following Elbrecht et al. (2016).

The manipulative period began on 22 October 2018 (Day 0). Nutrient enrichment (NaNO_3_ and KH_2_PO_4_) was continuously injected into half the mesocosms using a fluid‐metering pump (CK15, Kamoer Fluid Tech Co., Ltd. China) and pressure compensating drippers (model RXLD2SC; RX Plastics, New Zealand). Achieved concentrations were 2.19 ± 0.09 [mean ± *SE*] mg/L, N‐NO_3_^−^ and 0.12 ± 0.005 mg/L P‐PO_4_^+^ in the enriched treatment, compared to 0.57 ± 0.02 mg/L N‐NO_3_^−^ and 0.01 ± 0.001 mg/L P‐PO_4_^+^ in the ambient treatment (*n* = 192, measured on Days 1, 8, 15 and 18; only 32 mesocosms remained on the last two sampling occasions). Enrichment levels were chosen to remain within the “nutrient‐enriched” categories of the 6‐class water quality classification (GB 3838‐2002) of the Chinese Ministry of Environmental Protection (MEP, 2002), while also representing recognizably enriched levels according to other countries’ frameworks (European Environment Agency, 2015).

Fine sediment, collected from a dry floodplain downstream of the system, was air‐dried for 1 week and then sieved (mesh size 0.5 mm, D_50_ = 411.6 μm, Bettersize BT‐2900) before being added on Day 0 to half the mesocosms. This resulted in 100% sediment cover on Day 1, consistent with high sediment cover levels observed in Yangtze tributaries (Liu et al., 2016), compared with 0% cover for the 32 mesocosms without sediment. Such abrupt inputs of fine sediment are quite characteristic of river sites near newly built‐up areas by humans or when river morphology is being adjusted in China (N.P.D. Juvigny‐Khenafou, personal observations). Due to the negligible amount of fine sediment in the stream feeding the mesocosms, no increase in fine sediment cover with time was observed in the sediment‐free treatments.

Flow velocity reduction was achieved by removing the inflow jets, widening the inlet diameter, and pointing the inlet straight downwards in half of the mesocosms. This maintained the same discharge while reducing flow velocity below the detection limit of our instrument (0.02 m/s), thus avoiding confounding effects on nutrient concentrations and on unmanipulated physicochemical (e.g., water temperature, dissolved oxygen) and biological variables (e.g. drift of stream biota). Similar near‐bed flow velocities have been obtained in previous experiments using the same mesocosm system in other countries (New Zealand and Germany) where they resulted in considerable differences for the measured biological response variables (Beermann et al., 2018a; Bruder et al., 2016; Elbrecht et al., 2016).

### 2.3 Macroinvertebrate sampling

On each sampling occasion, water flow was stopped in two header tanks and the whole substratum and the associated benthic invertebrates of the 32 mesocosms were sieved in the field using a 150‐µm metal sieve and stored in 2‐L PET containers. These were immediately filled to the top with 95% ethanol and later stored at −18°C in the laboratory until processing. After ~12 hr, one third of the ethanol was replaced with fresh ethanol to account for any dilution caused by the water remaining in the substratum. In the laboratory, the invertebrates were elutriated with a 450‐µm sieve to remove the fine sediment and randomly divided into four equal subsamples. The specimens present in one subsample were counted, measured to the nearest 1 mm (body length excluding cerci and case; Piggott et al., 2015) and identified to family using a stereomicroscope (Leica EZ4HD 8‐35X, Leica microsystems GmbH, Germany), except for Nematoda, Oligochaeta, and Acari (Brooks et al., 2011). When specimens could not be confidently identified to a family, they were assigned to an order. We adopted this conservative approach across the whole dataset to reduce misassignments associated with the small size and general state of some specimens. Further, previous experiments suggest that family level of identification can be reliably used to examine community–environment and trait–environment relationships in aquatic habitats (Brooks et al., 2011; Tolonen et al., 2017). Adult Coleoptera families and Dipteran pupae were kept as individual taxa as they present different biological characteristics from their larval counterparts. The remaining 3/4 of each sample was scanned for rare taxa, which were added to the total taxon count in each sample. We then extrapolated the total invertebrate abundance for all taxa in each mesocosm by multiplying the subsample counts by 4.

We measured 27 benthos‐specific response variables: (a) total benthic invertebrate abundance, (b) benthic taxon richness, (c) Shannon's diversity index (H) to account for taxon richness in relation to their abundance, (d) Pielou's evenness index (J) to characterize how different in numbers invertebrate taxa were within each mesocosm community, (e) benthic EPT richness (number of taxa in the orders Ephemeroptera, Plecoptera and Trichoptera), (f) benthic EPT abundance, (g) three invertebrate size categories (following Piggott et al., 2015, Table 1), (h) multivariate community composition (Pillai's Trace statistic) based on the common taxa, and (i) individual abundances of the 17 most common benthic taxa, which represented 95.1% of all individuals when combined. We defined taxa as being common if they represented 0.3% or more of all individuals and were present in at least 50% of all mesocosms (*n* = 32) (Beermann et al., 2018a; Elbrecht et al., 2016). Alpha diversity metrics were calculated using the *vegan* R package (Oksanen et al., 2015).

**TABLE 1:** Functional trait classification of the benthic macroinvertebrates in the mesocosms and ecosystem functions known to be associated with each trait

Traits	Categories	Ecosystem functions
Lifecycle		
Reproductive cycle	Semivoltine Univoltine Multivoltine	Secondary productivity (Baxter et al., 2005; Benke, 2010; Gratton & Zanden, 2009)
Mobility		
Habit	Burrowers Crawlers Clingers Swimmers	Connectivity (Tonkin et al., 2018; Townsend & Hildrew, 1994)
Morphology		
Body shape	Streamlined Not streamlined	Stream metabolism (Brey, 2010; Burger et al., 2019; Hirst et al., 2014)
Maximum adult size	Small (1–9 mm) Medium (9–16 mm) Large (>16 mm)	
Respiration	Brachial Intertegumentary	
Foraging		
Functional feeding groups	Collector – gatherers Collector – filterers Scrapers Predators Shredders	Organic matter decomposition, nutrient cycling, water purification, primary and secondary production (Cummins, 2016; Grimm, 1988; Woodward et al., 2012)

### 2.4 Species trait data

All invertebrates were assigned into five trait groups, which were subsequently divided into 22 trait categories, using a binary code (Li et al., 2019). Adult beetles (which were rare) and insect pupae were excluded from this classification. Selected traits featured lifecycle, habit, functional feeding groups, morphology, and respiration strategy (Table 1). Together, these traits give an overall description of the ecological characteristics of the community and also represent aspects that are susceptible to having a close relationship with the manipulated stressors. Further, the traits provide information about the resilience and resistance of the community as well as more general biological characteristics (Ding et al., 2017; Dolédec et al., 2011; Li et al., 2019). Trait information was adapted from the literature (Ding et al., 2017; Merritt et al., 2008; Poff et al., 2006) and online databases (Appendix S2) (Schmidt‐Kloiber & Hering, 2015). A summary of the different trait categories can be found in Table 1.

We used the *dbFD* function in the FD R package (Laliberté & Legendre, 2010; Laliberté et al., 2014) to calculate functional richness, functional evenness, and functional divergence, as proposed by Villéger et al. (2008). Together, these metrics can indicate whether species in an environment are performing similar (i.e., redundant) or different (i.e., complementary) roles for a given function or service (Wilkinson et al., 2018). Functional richness measures the amount of the functional trait space filled by a given macroinvertebrate assemblage (i.e., the set of species found in each mesocosm) irrespective of the species’ abundances. Functional evenness quantifies the community's evenness in the functional trait space, whereas functional divergence measures the spread, that is, divergence in distribution, of species relative to the centroid of the functional trait space (Chevalier et al., 2019). Finally, we also determined functional redundancy as the difference between the Simpson's diversity and Rao's quadratic entropy (Wu et al., 2019). Functional redundancy characterizes the number of taxonomically distinct species that exhibit similar ecological functions (Wilkinson et al., 2018).

We constructed a site × trait abundance matrix to represent community functional structure for each sampling unit. This matrix is obtained by multiplying a species × trait matrix (see Appendix S1) by a site × species relative abundance matrix (Li et al., 2019). Only widespread trait categories occurring in at least 50% of all mesocosms were retained in this matrix to avoid introducing too many zero values. In total, we measured 24 trait‐specific variables: (a) functional richness, (b) functional evenness, (c) functional divergence, (d) functional redundancy, multivariate trait composition in the community (Pillai's Trace statistic), and (e) 19 trait categories.

### 2.5 Statistical analysis

All statistical analyses were performed using R (version 3.5.2, R Core Team). Where necessary, data were log‐transformed to improve normality and heteroscedasticity after exploratory data analysis. As in Juvigny‐Khenafou et al. (2020), we used a linear four‐factor model (ANOVA) with the following structure for all univariate analyses (all community‐level response variables): intercept (*df* 1) + nutrients (1) + sediment (1) + velocity (1) + time (1) + nutrients × time (1) + sediment × time (1) + velocity × time (1) + nutrients × sediment (1) + nutrients × velocity (1) + sediment × velocity (1) + nutrients × sediment × time (1) + nutrients × velocity × time (1) + sediment × velocity × time (1) + nutrients × sediment × velocity (1) + nutrients × sediment × velocity × time (1) + error (48; *n* = 64). The multivariate equivalent (MANOVA) of this model was used for the 17 common benthic taxa and the 19 widespread trait categories.

The significance level was set at *p* < 0.05, and all response patterns summarized in the Results were statistically significant unless indicated otherwise. Standardized effect sizes (partial *η* ^2^ values, range 0–1; Garson, 2015) are presented for *p*‐values < 0.1 to allow evaluating the likely biological relevance of the results (Nakagawa, 2004). After Nakagawa and Cuthill (2007), effect sizes can be classified as follows: <0.10 very small, ≥0.10 small, ≥0.30 medium, and ≥0.50 large. When evaluating factor main effects in the presence of interactions, we interpreted main effects (and lower‐order interactions) only where the effect size of the interaction was smaller than the size of the corresponding main effects (Quinn and Keough, 2002).

## 3 RESULTS

### 3.1 Community‐level metrics

Total invertebrate abundance decreased with fine sediment addition. Abundance also decreased with flow velocity after 2 weeks of stressor exposure but not after 3 weeks (velocity × time interaction) (Table 2, Figure S1). Total EPT abundance was likewise negatively affected by sediment addition and flow velocity reduction (Table 2). Total invertebrate taxon richness was unaffected by all treatments, whereas EPT taxon richness decreased when sediment was added. Lastly, Shannon's diversity decreased when sediment was added and Pielou's evenness showed a complex 3‐way interaction (nutrients × sediment ×flow velocity), with evenness being highest in nutrient‐enriched mesocosms with reduced flow velocity but no added sediment (Table 2, Figure S2).

**TABLE 2:** Summary (*p*‐values and effect sizes) of linear model results for macroinvertebrate community‐level response variables

Response	Nutrients	Sediment	Flow	Time	Nutrients × Sediment	Nutrients × Flow	Sediment × Flow	Nutrients × Time	Sediment × Time	Flow × Time	Nutrients × Sediment × Flow	Nutrients × Sediment × Time	Nutrients × Flow × Time	Sediment × Flow × Time	Nutrients × Sediment × Flow × Time
Total invertebrate abundance	0.40	**<0.001** (0.40) −	**0.01** (0.12) −	0.29	0.83	0.07 (0.07)	0.92	0.43	0.12	**0.01** (0.13)	0.08 (0.06)	0.66	0.79	0.35	0.33
Total EPT abundance	0.64	**<0.001** (0.50) −	**<0.001** (0.27) −	0.25	0.56	0.31	0.055 (0.07)	0.52	0.37	0.22	0.31	0.26	0.77	0.69	0.27
Taxon richness	0.86	0.055 (0.07)	0.50	0.93	1.00	0.55	0.18	0.40	0.45	0.73	0.67	0.73	0.93	1.00	0.21
EPT richness	0.89	**0.007** (0.14) −	0.89	0.67	1.00	0.89	0.78	0.67	0.78	0.67	0.57	1.00	0.33	0.78	0.053 (0.07)
Shannon's Diversity Index	0.30	**0.02** (0.11) −	0.94	0.62	1.00	0.92	0.16	0.25	0.81	0.73	0.07 (0.07)	0.73	0.99	0.54	0.67
Pielou's Evenness Index	0.21	0.15	0.52	0.54	0.94	0.53	0.46	0.34	0.32	0.38	**0.01** (0.14)	0.88	1.00	0.50	0.56
Small (<1 mm)	0.104	**0.03** (0.10) −	0.13	**0.01** (0.12) +	0.49	0.32	0.56	**0.03** (0.09)	0.16	**0.008** (0.14)	0.18	0.11	0.68	0.84	0.30
Medium (1–5 mm)	0.32	**<0.001** (0.34) −	**0.02** (0.11) −	0.46	0.85	0.097 (0.06)	0.68	0.74	0.25	0.06 (0.07)	0.16	0.84	0.74	0.43	0.70
Large (>5 mm) (log10)	**0.005** (0.15) +	**<0.001** (0.30) −	0.87	**0.006** (0.14) −	0.44	0.46	0.37	**0.04** (0.08)	0.85	0.94	0.47	0.40	0.63	0.07 (0.07)	0.089 (0.06)

Note

For all manipulated factors, main effects are classified directionally as positive (+) or negative (−).*p*‐values are in bold font where *p* < 0.05. Effect sizes (partial‐*η* ^2^ values; range 0–1) are shown in parentheses for all cases where *p* < 0.10. Total invertebrate count = 37,244.

### 3.2 Body size metrics

**FIGURE 2:**
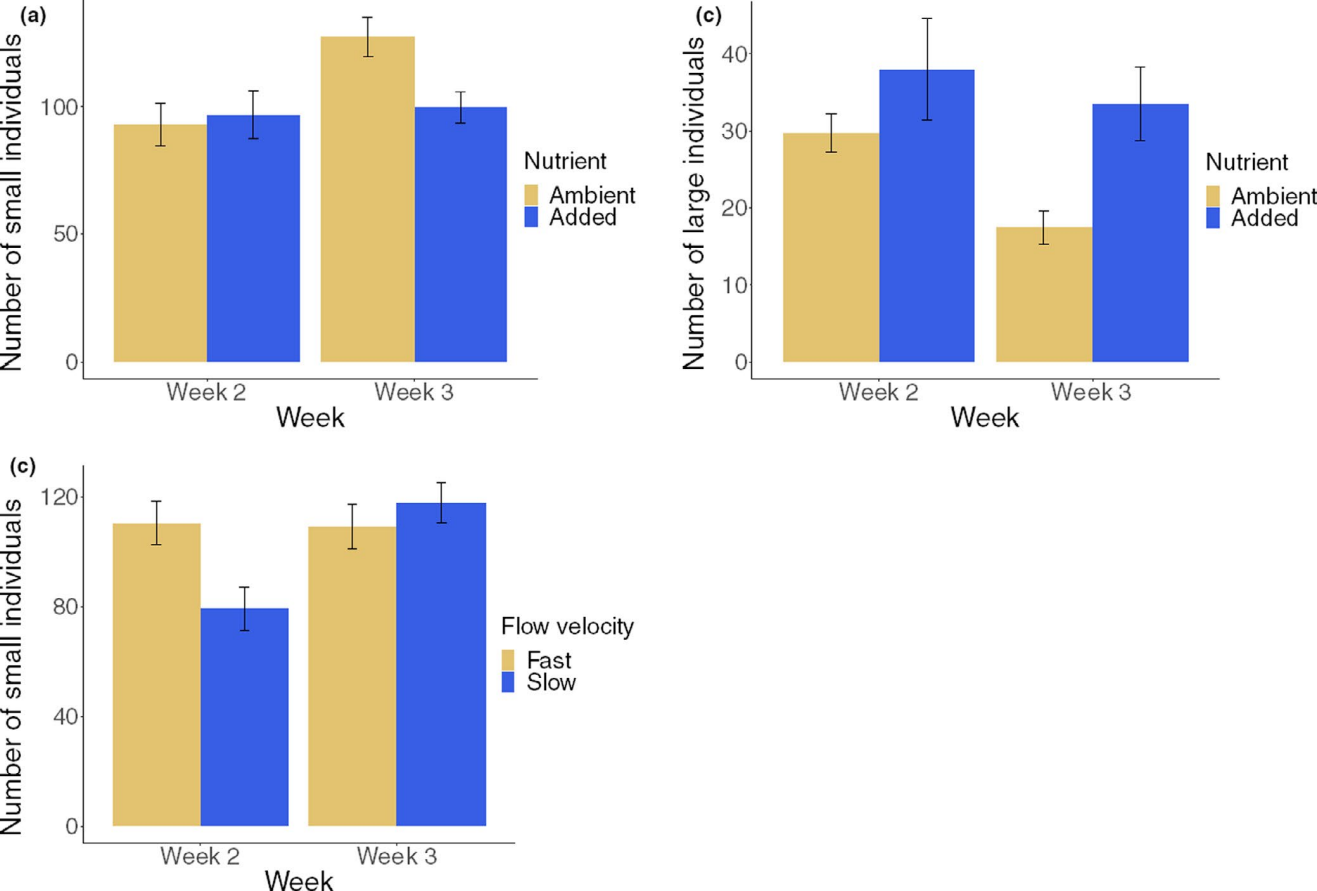
Average numbers per mesocosm of small or large benthic macroinvertebrates on the two sampling occasions, showing the main effects of flow velocity reduction and nutrient enrichment (error bars = ±*SE*, *n* = 32 per treatment)

Abundances of invertebrates in all three size categories decreased with sediment addition (Table 2). The effect of nutrient enrichment on small invertebrates (<1 mm) changed from neutral after 2 weeks of stressor exposure to negative after 3 weeks (Figure 2a), preventing the increase of small individuals with time observed in the ambient treatment. By contrast, nutrient enrichment increased abundance of large invertebrates (>5 mm) on both sampling dates; moreover, fewer large individuals were found after 3 weeks than after 2 weeks (Figure 2b, Table 2). The effects of flow velocity reduction on small invertebrates were negative after 2 weeks but positive after 3 weeks (Figure 2c). Finally, medium‐sized invertebrates (1–5 mm) became rarer when flow velocity was reduced (Table 2).

### 3.3 Multivariate community composition and individual common taxa

The multivariate results of our analysis indicated that invertebrate community composition changed in response to added sediment and when the three stressors were manipulated together; community composition also changed from week 2 to week 3 (Table 3). Regarding taxon‐specific responses, 41% of the abundant taxa (seven of 17) responded to at least one experimental factor as a stressor main effect. All these seven taxa (the mayfly families Heptageniidae, Baetidae and Ephemerellidae, the dipterans Chironomidae and Tipulidae, the caddisfly family Leptoceridae, and the stonefly family Nemouridae) were affected by added sediment, followed by flow velocity reduction (Heptageniidae, Baetidae) and nutrient enrichment (Nemouridae). These ten stressor main effects were all negative (Table 3). Changes with time (independent of stressor effects) occurred for two taxa; Heptageniidae became generally more abundant after 3 weeks of stressor exposure whereas dipteran pupae became generally rarer.

**TABLE 3:** Summary (*p*‐values and effect sizes) of multi‐ and univariate linear model results for the common taxa (with relative abundances)

Response	Nutrients	Sediment	Flow	Time	Nutrients × Sediment	Nutrients × Flow	Sediment × Flow	Nutrients × Time	Sediment × Time	Flow × Time	Nutrients × Sediment × Flow	Nutrients × Sediment × Time	Nutrient × Flow × Time	Sediment × Flow × Time	Nutrients × Sediment × Flow × Time
**Community (95%)**	0.45	**<0.001** (0.70)	0.06 (0.50)	**0.02** (0.55)	0.93	0.15	0.18	0.56	0.25	0.39	**0.02** (0.55)	0.13	0.44	0.98	0.97
Heptageniidae (6.9%)	0.17	**<0.001** (0.38) −	**0.02** (0.10) −	**0.03** (0.09) +	0.37	0.49	0.08 (0.06)	0.98	0.52	0.86	0.37	0.40	0.49	0.90	0.37
Baetidae (21.1%)	0.96	**<0.001** (0.36) −	**<0.001** (0.38) −	0.71	0.37	0.48	**0.046** (0.08)	0.60	0.08 (0.06)	0.17	0.37	0.21	0.60	0.56	0.35
Ephemerellidae (4.3%)	0.48	**<0.001** (0.32) −	0.96	0.15	0.22	**0.04** (0.08)	0.70	**0.01** (0.12)	0.12	0.09 (0.05)	0.96	0.09	0.12	0.87	0.96
Caenidae (1.2%)	1.00	0.10	0.24	0.24	0.48	0.81	0.64	0.24	**0.02** (0.10)	**0.02** (0.10)	0.64	0.16	0.06 (0.07)	0.81	0.10
Leptophlebiidae (0.6%)	0.24	0.15	0.38	0.15	0.56	0.25	0.38	0.56	0.77	0.77	0.56	0.25	0.56	0.77	1.00
Chironomidae (48.1%)	0.17	**0.01** (0.13) −	0.76	0.24	0.45	0.12	0.39	0.88	0.12	**0.01** (0.12)	**0.01** (0.11)	0.98	0.58	0.23	0.56
Empididae (0.4%)	0.71	1.00	0.46	0.46	1.00	0.46	0.27	0.46	0.27	0.27	0.27	0.07 (0.07)	0.71	1.00	1.00
Tipulidae (0.6%)	0.73	**0.04** (0.08) −	0.31	0.18	0.18	0.73	1.00	0.18	**0.02** (0.10)	**0.04** (0.08)	0.18	0.73	1.00	0.73	0.31
Dipteran pupae (1.9%)	0.96	0.63	0.42	**0.01** (0.13) −	0.63	0.55	**0.03** (0.09)	0.26	0.12	0.55	0.55	0.63	0.55	0.42	0.42
Leptoceridae (1.7%)	0.40	**0.02** (0.10) −	0.61	0.50	0.18	0.13	0.74	0.18	0.87	0.18	0.50	0.07 (0.07)	0.50	0.24	0.87
Hydroptilidae (0.5%)	0.49	0.07 (0.06)	0.71	0.28	1.00	0.47	0.72	0.47	0.72	0.72	0.47	1.00	1.00	0.72	0.15
Elmidae (3.5%)	0.84	0.61	0.54	0.10	1.00	0.36	0.54	0.61	0.84	0.26	0.61	0.76	0.15	0.47	0.54
Perlidae (0.4%)	0.75	0.34	0.75	0.21	0.53	1.00	0.53	0.34	0.34	0.34	0.75	0.21	0.53	0.53	0.75
Nemouridae (0.6%)	**0.01** (0.12) −	**0.03** (0.09) −	0.39	0.60	0.60	0.23	**0.01** (0.12)	0.12	0.60	0.39	0.86	0.39	0.23	0.86	0.86
Acari (0.5%)	0.26	0.45	0.26	0.26	0.70	0.14	0.26	0.45	0.26	0.45	1.00	0.45	0.06 (0.07)	0.45	0.70
Gordiidae (0.5%)	0.29	0.14	0.06 (0.07)	0.83	0.29	0.83	0.06 (0.07)	0.53	0.53	0.83	**0.02** (0.10)	0.29	0.53	0.83	0.14
Nematoda (1.9%)	0.62	0.67	0.92	0.72	0.92	0.62	0.97	0.31	0.62	0.31	0.20	0.20	0.67	0.97	0.62

Note

Community *p*‐values are for the Pillai's Trace statistic. For all manipulated factors, significant main effects are classified directionally as positive (+) or negative (−). *p*‐values are in bold font where *p* < 0.05. Effect sizes (partial‐*η* ^2^ values; range 0–1) are shown in parentheses for all cases where *p* < 0.1. Total invertebrate count = 37,244.

**FIGURE 3:**
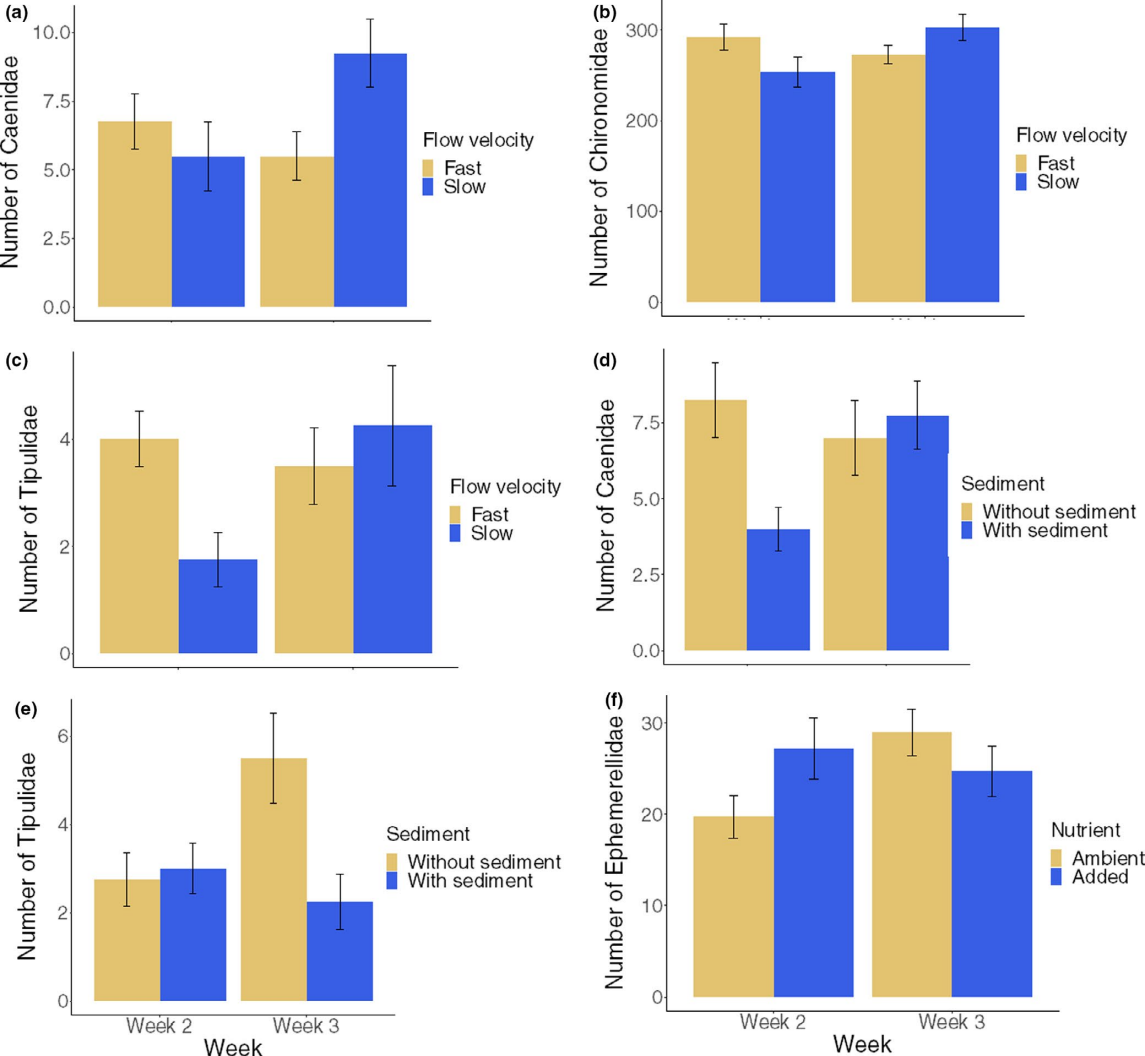
Average numbers of common invertebrate taxa affected by stressor main effects across both sampling occasions (error bars = ±*SE*, *n* = 16)

Temporal changes in stressor main effects affected four taxa and occurred in six cases (three for flow velocity, two for sediment and one for nutrients) (Figure 3a–f). Caenidae, Chironomidae, and Tipulidae all decreased in abundance after 2 weeks of exposure to flow velocity reduction (Figure 3a–c). However, after 3 weeks their populations seemed to have adjusted and increases in abundance were observed for all three taxa. For Caenidae, sediment decreased abundance 2 weeks after addition but this negative effect had disappeared after 3 weeks (Figure 3d). The opposite temporal response pattern to sediment was observed for Tipulidae, where sediment addition prevented the increase with time observed in the sediment‐free treatment (Figure 3e). Finally, Ephemerellidae increased in abundance when nutrients were added after 2 weeks, whereas after 3 weeks, this effect had been reversed (Figure 3f).

Interactions among stressors were almost as common as stressor main effects, affecting six common taxa (35%). Sediment × flow velocity interactions occurred for Baetidae, Nemouridae, and dipteran pupae, nutrients × flow velocity interacted when affecting Ephemerellidae, and complex nutrients × sediment × flow velocity interaction occurred for Chironomidae and Gordiidae worms (Figures S7–S8).

### 3.4 Functional diversity and traits

**FIGURE 4:**
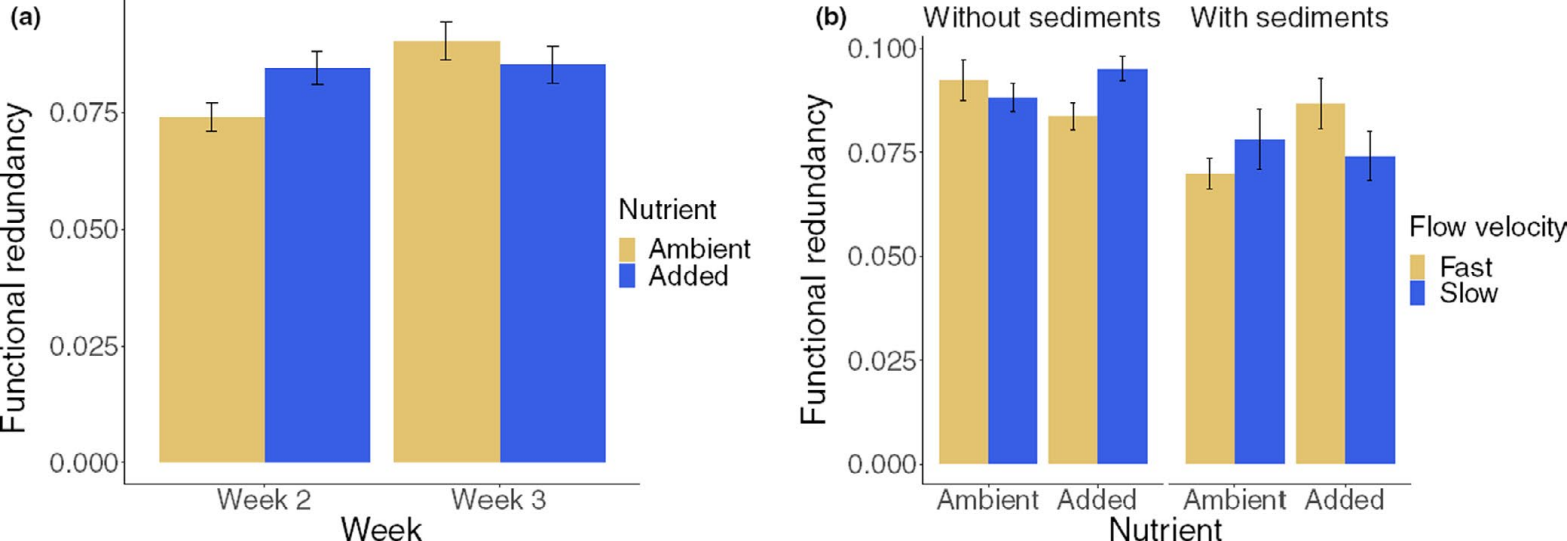
Nutrient main effects across sampling dates and three‐stressor plot (averaged across both dates) for functional redundancy (error bars = ±*SE*)

Two of the four functional diversity metrics were affected by sediment as a main effect, while none showed main effects for flow velocity reduction or nutrient enrichment. Functional evenness increased when sediment was added whereas functional redundancy declined (Table 4). Nutrients increased functional redundancy after 2 weeks of enrichment but not after 3 weeks (Figure 4a, Table 4), and redundancy also increased with time regardless of the stressor treatments. Further, functional redundancy showed a complex three‐stressor interaction (Figure 4b). Nutrient addition increased functional redundancy at fast flow combined with added sediment but decreased redundancy at fast flow without sediment, whereas the opposite patterns occurred at slow flow.

**TABLE 4:** Summary (*p*‐values and effect sizes) of linear model results of the functional diversity measurements

Response	Nutrients	Sediment	Flow	Time	Nutrients × Sediment	Nutrients × Flow	Sediment × Flow	Nutrients × Time	Sediment × Time	Flow × Time	Nutrients × Sediment × Flow	Nutrients × Sediment × Time	Nutrients × Flow × Time	Sediment × Flow × Time	Nutrients × Sediment × Flow × Time
FRic (log10 + 1)	0.39	0.185	0.45	0.81	0.97	0.71	0.19	0.76	0.50	0.91	0.98	0.94	0.55	0.68	0.15
FEve	0.12	**<0.001** (0.24) +	0.33	0.42	0.99	0.29	0.17	0.62	0.62	0.1003	0.92	0.41	0.14	0.98	0.44
FDiv	0.82	0.09 (0.06)	0.37	0.41	0.86	0.88	0.73	0.54	0.73	0.68	0.13	0.65	0.17	0.47	0.68
FR	0.39	**<0.001** (0.25) −	0.81	**0.01** (0.13) +	0.26	0.69	0.36	**0.02** (0.11)	0.78	0.20	**0.001** (0.14)	0.65	0.91	0.11	0.39

Note

FRic = Functional richness; FEve = Functional evenness; FDiv = Functional divergence; FR = Functional redundancy. For all manipulated factors, main effects are classified directionally as positive (+) or negative (−). *p*‐values are bolded where *p* < 0.05. Effect sizes (partial‐*η* ^2^ values; range 0–1) are shown in parentheses for all cases where *p* < 0.1.

The multivariate results of the trait analysis indicated that trait community composition (based on the 19 widespread trait categories) changed in response to sediment addition (Table 5); further, trait composition changed from week 2 to week 3 independently of the stressor treatments. Sediment (10 trait categories affected) and flow velocity (9) were the most pervasive stressors, followed by nutrients (2) (Table 5). Semivoltinism, Multivoltinism, burrowing and crawling mobilities, predation, tegumentary respiration and non‐streamlined morphology were favored in mesocosms with added sediment whereas medium adult size, brachial respiration and streamlined bodies became less prevalent. Flow velocity reduction favored univoltinism, borrowing and crawling mobilities, predation, tegumentary respiration, and non‐streamlined morphology. The opposite was found for swimmers, streamlined morphology, and brachial respiration. Flow velocity reduction also triggered the single change across sampling dates observed in the trait dataset, with a positive influence on the abundance of shredders occurring only after the first 2 weeks of exposure to this stressor (Figure S10). Finally, nutrient enrichment favored the settlement of individuals with a large maximum adult body size while reducing the smaller ones. Interactions between stressors were not very frequent (15.7%) but all involved flow velocity reduction (Figures S11–S13).

**TABLE 5:** Summary (*p*‐values and effect sizes) of multi‐ and univariate linear model results for the widespread trait categories (with percentage across all samples)

Response	Nutrient	Sediment	Flow	Time	Nutrient × Sediment	Nutrient × Flow	Sediment × Flow	Nutrient × Time	Sediment × Time	Flow × Time	Nutrient × Sediment × Flow	Nutrient × Sediment × Time	Nutrient × Flow × Time	Sediment × Flow × Time	Nutrient × Sediment × Flow × Time
**Trait community** **(MANOVA)**	0.053 (0.55)	**0.03** (0.58)	**0.17**	**<0.001** (0.74)	0.83	0.87	0.50	0.51	0.15	0.16	0.63	0.35	0.78	0.86	0.65
Semivoltine (4.4%)	0.46	**0.04** (0.09) +	0.51	**0.006** (0.15) −	0.80	0.72	0.86	0.90	0.14	0.59	0.65	0.54	0.23	1.00	0.48
Univoltine (6.8%)	0.41	0.27	**<0.001** (0.24) +	0.17	0.23	0.57	0.64	0.36	0.11	0.77	0.20	0.66	0.19	0.74	0.62
Multivoltine (73.3%)	0.49	**0.01** (0.12) +	0.66	0.55	0.80	073	0.51	0.50	0.052 (0.08)	0.31	0.36	0.99	0.37	0.39	0.84
Burrower (50.4%)	0.71	**<0.001** (0.26) +	**0.003** (0.17) +	0.96	0.46	0.63	0.48	0.34	0.70	0.33	0.09 (0.05)	0.56	0.97	0.44	0.52
Crawler (64.8%)	0.79	**<0.001** (0.21) +	**<0.001** (0.25) +	0.35	0.72	0.86	0.096 (0.06)	0.65	0.81	0.54	0.89	0.77	0.95	0.59	0.67
Clinger (89.4%)	0.43	0.51	0.08 (0.06)	0.20	0.53	0.63	0.07 (0.07)	0.25	0.59	0.57	0.15	0.84	0.95	0.79	0.94
Swimmer (25.8%)	0.80	0.051 (0.08)	**<0.001** (0.24) −	0.77	0.14	0.65	0.07 (0.06)	0.59	0.12	0.84	0.37	0.51	0.59	0.67	0.52
Streamlined (30.3%)	0.68	**<0.001** (0.22) −	**<0.001** (0.28) −	0.46	0.51	0.62	**0.008** (0.14)	0.72	0.69	0.35	0.84	0.76	0.77	0.76	0.47
Not streamlined (67%)	0.70	**<0.001** (0.22) +	**<0.001** (0.31) +	0.99	0.58	0.62	**0.04** (0.08)	0.93	0.99	0.34	0.81	0.68	0.68	0.83	0.41
Small (86.3%)	**0.03** (0.09) −	0.53	0.11	0.13	0.98	0.63	0.08 (0.06)	0.66	0.49	0.58	0.26	0.21	0.17	0.94	0.65
Medium (18.8%)	0.65	**0.001** (0.20) −	0.74	0.17	0.51	0.85	0.61	0.91	0.24	0.45	0.08 (0.06)	0.63	0.11	0.60	0.59
Large (1.7%)	**0.02** (0.10) +	0.63	0.22	0.48	0.98	0.33	0.92	0.89	0.41	0.73	0.91	0.83	0.85	0.66	0.11
Gatherer (81.9%)	0.78	0.106	0.77	0.55	0.53	0.91	0.42	0.78	0.36	0.17	0.59	0.19	0.78	0.51	0.68
Filterer (0.8%)	0.08 (0.06)	0.90	0.86	0.34	0.65	0.67	0.056 (0.07)	0.69	0.25	0.70	0.32	0.98	0.88	0.59	0.33
Scraper (15.8%)	0.51	0.08 (0.06)	0.91	0.75	0.68	0.78	0.52	0.49	0.35	0.94	0.43	0.27	0.97	0.36	0.59
Predator (53.6%)	0.73	**<0.001** (0.29) +	**<0.001** (0.22) +	0.68	0.57	0.54	0.38	0.58	0.78	0.50	0.21	0.17	0.98	0.43	0.65
Shredder (3.2%)	0.41	0.34	0.09 (0.06)	**0.004** (0.13) −	0.20	**0.02** (0.10)	0.34	0.72	0.35	**0.03** (0.08)	0.69	0.08 (0.06)	0.70	0.39	0.67
Brachial (44.1%)	0.80	**<0.001** (0.25) −	**0.01** (0.20) −	0.92	0.35	0.56	0.07 (0.07)	0.98	0.52	0.35	0.32	0.25	0.87	0.48	0.45
Tegumentary (53.9%)	1.00	**<0.001** (0.27) +	**<0.001** (0.26) +	0.79	0.38	0.48	0.24	0.75	0.86	0.44	0.29	0.18	0.78	0.59	0.39

Note

Trait community *p*‐values are for the Pillai's Trace statistic. For all manipulated factors, significant main effects are classified directionally as positive (+) or negative (−). *p*‐values are in bold font where *p* < 0.05. Effect sizes (partial‐*η* ^2^ values; range 0–1) are shown in parentheses for all cases where *p* < 0.1.

## 4 DISCUSSION

### 4.1 Stressor main effects on the invertebrate community

We found sedimentation and flow velocity reduction to have the most pervasive effects on stream macroinvertebrate community assemblage and trait composition, as predicted in our first hypothesis. All observed effects of both these stressors on invertebrate community‐level metrics and abundances of common taxa were negative. This result differs from previous experiments involving the same stressors, perhaps due to the fact that the source community for this experiment came from a “near‐pristine” stream compared to agricultural streams in Germany, Ireland, or New Zealand (Beermann et al., 2018a; Davis et al., 2018; Elbrecht et al., 2016; Piggott et al., 2015). Fine sediment deposition caused a decrease in total invertebrate abundance irrespective of invertebrate size categories. We attribute this response to habitat homogenization (Petsch et al., 2017), decrease in food availability (Matthaei et al., 2010), and physical damage to the breathing apparatus of gilled invertebrates (Piggott et al., 2015; Wagenhoff et al., 2012; Wood & Armitage, 1997). The likely detrimental effect on brachial respiration is further supported by our univariate trait analysis, which displayed an increase in integumentary respiration concomitant to a decrease in brachial respiration when sediment was added. Further, abundance and richness of EPT taxa decreased with sediment addition overall, which was also reflected in the individual taxon responses. The lower abundances of these taxa in mesocosms with added fine sediment are likely due to increased emigration rates via drift and/or emergence (Beermann et al., 2018a; Piggott et al., 2015).

Flow velocity reduction was the second‐most pervasive stressor and displayed the largest number of changes with time in its effects on invertebrate community‐level metrics and abundances of common taxa. Thus, the negative effect of flow velocity reduction after 2 weeks of stressor exposure on total invertebrate abundance and abundances of small individuals Chironomidae, Tipulidae, and Caenidae was no longer observed after 3 weeks. A similar situation was also observed in the response of Caenidae to sediment addition. We suggest that interspecific microhabitat preference differences within these families led to an increased short‐term drift response to reduced flow velocity (and sediment addition for Caenidae), which was later masked by recolonization of individuals within the same families that can tolerate or prefer slow flows (Harding et al., 2019; Zhang & Malmqvist, 1997). Previous studies have shown that flow velocity reduction often increases drift propensity, especially of swimming taxa (Beermann et al., 2018a; Piggott et al., 2015), which is supported by our finding of fewer swimming taxa at reduced flow velocity (see *Stressor main effects on functional diversity and trait categories* below). Further mesocosm experiments involving drift sampling should be done to confirm these patterns (Beermann et al., 2018a).

Our second hypothesis—enhancement of the biomass accumulation potential in response to nutrient enrichment either via increased carrying capacity or a shift toward larger‐bodied organisms—was largely supported. Abundance of large‐bodied individuals increased in nutrient‐enriched mesocosms whereas small‐sized organisms became rarer after 3 weeks of enrichment, despite total invertebrate abundance and community composition being similar (except for Nemouridae). Because immigration rates by drift into the mesocosms can be expected to be similar for all mesocosms (see Magbanua et al., 2013), this suggests invertebrate in nutrient‐enriched mesocosms grew faster, with small individuals becoming medium‐sized and medium‐sized ones becoming large (Frost & Elser, 2002). Based on the abundances of small, medium, and large individual over time, we postulate the influx and outflux of medium‐sized invertebrates more or less evened out, whereas outflux of large individuals exceeded the influx. Further, individual trait information indicates that nutrient‐enriched mesocosms harbored taxa with a larger maximum adult body size, supporting the idea that moderate nutrient enrichment acted as a subsidy, favoring the growth of macroinvertebrates with higher metabolic requirements (Cross et al., 2003). Based on a related experiment by Wagenhoff et al. (2012), our moderate level of nutrient enrichment was probably already past the subsidy thresholds for abundances of EPT and Chironomidae, two of the most abundant invertebrate groups in our experiment. Thus, we attribute the observed nutrient effects in our system to faster invertebrate growth rates combined with a capacity to support larger organisms rather than an increase in total carrying capacity.

### 4.2 Stressor main effects on functional diversity and trait categories

In agreement with previous studies, sedimentation and flow velocity reduction were key stressors driving functional diversity and trait category responses (Buendia et al., 2013; Calapez et al., 2018). However, because the colonizing invertebrate species pool was the same for all experimental units and total taxon richness remained similar across all stressor treatments, it is not surprising that functional richness and dispersion were also unaffected by our treatments. Traits were neither “lost” nor “gained”, but rather relative abundances were rearranged to reflect changes in the dominance patterns of the taxa best adapted to the new environmental conditions. Consequently, the reduction in functional redundancy associated with sedimentation can probably be attributed to a smaller density of individuals performing the same functions. This suggests that the stress‐induced community may be more vulnerable to further “functional loss” (Cummins, 2016; Pillar et al., 2013). Additionally, nutrient enrichment increased functional redundancy and seemed to dampen changes with time, probably by allowing species with similar resource needs to coexist via an increase in quantity and quality of resources (Piggott et al., 2015; Sterner et al., 1993).

Shifts in feeding behaviors were also observed, with an increase in the relative abundances of predatory species when sediment was added or current velocity reduced. This result differs from Rabení et al. (2005) who reported a decline in total predator density when fine sediment cover increased, although these authors observed a broad predatory taxon‐specific tolerance spectrum linked to their mobility. Our system lacked higher‐order predators; therefore, it is possible that sediment deposition favored individuals capable of burrowing or crawling without becoming prey themselves (Ding et al., 2017; Li et al., 2019; Rabení et al., 2005). Further, we speculate that reduced hiding space due to sediment deposition filling interstitial spaces in the mesocosm beds, combined with an increased mobility of crawling predators, most likely facilitated their prey‐catching success rate, which could explain their increased density under these conditions. We also observed an increase in the relative abundance of shredders after 2 weeks of reduced flow velocity, which could be related to an increase in CPOM retention with velocity reduction (Death et al., 2009). In our experimental setup, CPOM variations are highly dependent on CPOM load fluctuations in the stream feeding the setup; these were not recorded but could help explain the temporal pattern observed for shredders. When combined, the observed effects of sediment addition and reduced flow velocity on the invertebrate trait variables suggest a facilitation of secondary productivity under these conditions.

Interestingly, trait responses to added fine sediment and reduced current velocity were often concomitant. For example, we observed a reduction in streamlined individuals, while the opposite response occurred for non‐streamlined individuals associated with an increase in burrowing and crawling individuals in both sedimented and reduced flow velocity mesocosms. Slower flow velocities facilitate sedimentation of fine particles but also make it easier for less hydrodynamic organisms to move around. This suggests that, in our experiment, taxa adapted to reduced velocities also usually possessed features associated with increased sedimentation and vice versa.

### 4.3 Interactive effects on community and functionality

Interactions between two or all three manipulated stressors affected six of 17 common taxa and three of 19 widespread trait categories. Five of these interactions occurred between flow velocity and sediment, two between flow velocity and nutrients, and two were interactions between all three stressors, thus partially supporting our third hypothesis that interactions involving nutrient enrichment should be least common. Previous experiments have highlighted the importance of flow velocity and sediment deposition in shaping stream invertebrate community structure and functionality (Buendia et al., 2013; Dolédec et al., 2011; Elbrecht et al., 2016). Even though nutrient enrichment appeared to be relatively less important in shaping invertebrate responses in our experiment, past studies have shown that the effects of nutrient enrichment can differ strongly along an increasing gradient of concentration (Wagenhoff et al., 2012). Our enrichment treatment was fairly low compared to some previous similar experiments (Elbrecht et al., 2016); thus, it was perhaps not high enough (or long enough, at only 3 weeks of enrichment) to trigger strong responses of both community structure and trait composition. On the other hand, our enrichment might have already exceeded the subsidy threshold of our mesocosm ecosystem, resulting in a decline in many response variables compared to their peak subsidy enrichment point (Wagenhoff et al., 2012). Distinguishing between the two outcomes would require further work involving a finer scale of nutrient (N + P) enrichment, to identify which side of the subsidy‐stress gradient our results fall into.

In our datasets, interactive effects between stressors occurred most often in the abundance patterns of individual common taxa. Past experiments using the same stream mesocosm setup in Ireland and Germany also observed a similar trend (Davis et al., 2018; Elbrecht et al., 2016). In all three studies, moreover, EPT taxa were more sensitive than other taxa to two‐way interactive effects between flow velocity reduction and either nutrient enrichment or sedimentation, as one might expect according to their high sensitivity to environmental changes (Bonada et al., 2006). The only taxon‐specific three‐way interaction observed in our experiment was a negative response of Chironomidae when exposed to all three stressors simultaneously. This result may seem surprising because this family is usually considered to be fairly tolerant to agricultural and urbanization stressors (Li et al., 2019; Mor et al., 2019). However, we suspect this intricate three‐way interaction to stem from the complexity of the Chironomidae family, which encompasses a diverse range of genera and species that vary widely in their microhabitat preferences and tolerance of various stressors. Thus, it is possible that while some midge species were more tolerant to one or two stressors, the overall family responded negatively to all three stressors combined. These results lend more weight to the recommendation of Elbrecht et al. (2016) and Beermann et al. (2018a) that Chironomidae should be studied with a finer taxonomical resolution, for example by using DNA metabarcoding (Beermann et al., 2018b), to fully understand their response patterns to interacting anthropogenic stressors.

### 4.4 Conclusions

The present study shows how multiple‐stressor research can move beyond community assessments to anticipate changes in ecosystem stability and ecosystem processes in response to stressors. Our experiment demonstrates the complexity of macroinvertebrate community dynamics and individual taxon responses to multiple agricultural stressors. Although traits and functional diversity showed a higher proportion of stressor main effects (74% of functional variables affected compared to 58% for community/taxon variables), invertebrate community and taxon responses were more sensitive to stressor interactions (31% vs. 17%). Thus, taxonomical and trait approaches are highly complementary, even over short spatial and temporal scales (Cummins, 2016). While community abundance patterns can help us investigate macroinvertebrates dynamics, trait‐based approaches give a mechanical indication of the reasons why. Finally, functional diversity facilitates predictions about the stability of a given system when exposed to multiple stressors (Pillar et al., 2013). Further studies, ideally repeated over different seasons, spatial scales and incorporating ecosystem processes such as energy transfer between trophic levels, should be conducted to improve our knowledge of macroinvertebrate community responses to multiple stressors (Kardol et al., 2018).

## CONFLICT OF INTEREST

The authors declare no conflict of interest.

## AUTHOR CONTRIBUTIONS

**Noel P. D. Juvigny‐Khenafou:** Conceptualization (lead); data curation (lead); formal analysis (lead); investigation (lead); methodology (lead); project administration (lead); validation (lead); visualization (lead); writing – original draft (lead); writing – review and editing (equal). **Jeremy J. Piggott:** Conceptualization (lead); funding acquisition (lead); investigation (supporting); methodology (lead); project administration (supporting); supervision (lead); validation (supporting); writing – review and editing (supporting). **David Atkinson:** Funding acquisition (supporting); investigation (supporting); project administration (supporting); supervision (supporting); writing – original draft (supporting); writing – review and editing (supporting). **Yixin Zhang:** Conceptualization (supporting); data curation (supporting); formal analysis (supporting); funding acquisition (lead); investigation (supporting); methodology (supporting); project administration (equal); supervision (equal); writing – original draft (supporting); writing – review and editing (supporting). **Samuel J. Macaulay:** Conceptualization (supporting); data curation (supporting); methodology (supporting); writing – review and editing (supporting). **Naicheng Wu:** Formal analysis (supporting); project administration (supporting); supervision (supporting); validation (lead); writing – original draft (supporting); writing – review and editing (supporting). **Christoph D. Matthaei:** Formal analysis (lead); funding acquisition (lead); investigation (supporting); methodology (supporting); supervision (supporting); validation (lead); writing – original draft (lead); writing – review and editing (lead).

## Supporting information

Appendix S1

Appendix S2

## Data Availability

https://doi.org/10.6084/m9.figshare.11791287.

## References

[ece36979-bib-0001] Allan, J. D. (2004). Landscapes and riverscapes: The influence of land use on stream ecosystems. Annual Review of Ecology Evolution and Systematics, 35, 257–284. 10.1146/annurev.ecolsys.35.120202.110122

[ece36979-bib-0002] Baxter, C. V. , Fausch, K. D. , & Saunders, W. C. (2005). Tangled webs: Reciprocal flows of invertebrate prey link streams and riparian zones. Freshwater Biology, 50, 201–220. 10.1111/j.1365-2427.2004.01328.x

[ece36979-bib-0003] Beermann, A. J. , Elbrecht, V. , Karnatz, S. , Ma, L. , Matthaei, C. D. , Piggott, J. J. , & Leese, F. (2018a). Multiple‐stressor effects on stream macroinvertebrate communities: A mesocosm experiment manipulating salinity, fine sediment and flow velocity. Science of the Total Environment, 610–611, 961–971. 10.1016/j.scitotenv.2017.08.084 28830056

[ece36979-bib-0004] Beermann, A. J. , Zizka, V. M. A. , Elbrecht, V. , Baranov, V. , & Leese, F. (2018b). DNA metabarcoding reveals the complex and hidden responses of chironomids to multiple stressors. Environmental Sciences Europe, 30, 26 10.1186/s12302-018-0157-x

[ece36979-bib-0005] Benke, A. C. (2010). Secondary production as part of bioenergetic theory‐contributions from freshwater benthic science. River Research and Applications, 26, 36–44. 10.1002/rra.1290

[ece36979-bib-0006] Bonada, N. , Prat, N. , Resh, V. H. , & Statzner, B. (2006). Developments in aquatic insect biomonitoring: A comparative analysis of recent approaches. Annual Review of Entomology, 51, 495–523. 10.1146/annurev.ento.51.110104.151124 16332221

[ece36979-bib-0007] Brey, T. (2010). An empirical model for estimating aquatic invertebrate respiration. Methods in Ecology and Evolution, 1, 92–101. 10.1111/j.2041-210X.2009.00008.x

[ece36979-bib-0008] Brooks, A. J. , Chessman, B. C. , & Haeusler, T. (2011). Macroinvertebrate traits distinguish unregulated rivers subject to water abstraction. Journal of the North American Benthological Society, 30, 419–435. 10.1899/10-074.1

[ece36979-bib-0009] Bruder, A. , Salis, R. K. , McHugh, N. J. , & Matthaei, C. D. (2016). Multiple‐stressor effects on leaf litter decomposition and fungal decomposers in agricultural streams contrast between litter species. Functional Ecology, 30, 1257–1266. 10.1111/1365-2435.12598

[ece36979-bib-0010] Buendia, C. , Gibbins, C. N. , Vericat, D. , Batalla, R. J. , & Douglas, A. (2013). Detecting the structural and functional impacts of fine sediment on stream invertebrates. Ecological Indicators, 25, 184–196. 10.1016/j.ecolind.2012.09.027

[ece36979-bib-0011] Burger, J. R. , Hou, C. , & Brown, J. H. (2019). Toward a metabolic theory of life history. Proceedings of the National Academy of Sciences of the United States of America, 116, 26653–26661. 10.1073/pnas.1907702116 PMC6936346 31822607

[ece36979-bib-0012] Calapez, A. R. , Serra, S. R. Q. , Santos, J. M. , Branco, P. , Ferreira, T. , Hein, T. , Brito, A. G. , & Feio, M. J. (2018). The effect of hypoxia and flow decrease in macroinvertebrate functional responses: A trait‐based approach to multiple‐stressors in mesocosms. Science of the Total Environment, 637–638, 647–656. 10.1016/j.scitotenv.2018.05.071 29758421

[ece36979-bib-0013] Cardinale, B. J. , Duffy, J. E. , Gonzalez, A. , Hooper, D. U. , Perrings, C. , Venail, P. , Narwani, A. , MacE, G. M. , Tilman, D. , Wardle, D. A. , Kinzig, A. P. , Daily, G. C. , Loreau, M. , Grace, J. B. , Larigauderie, A. , Srivastava, D. S. , & Naeem, S. (2012). Biodiversity loss and its impact on humanity. Nature, 486, 59–67. 10.1038/nature11148 22678280

[ece36979-bib-0014] Chevalier, M. , Lindström, Å. , Pärt, T. , & Knape, J. (2019). Changes in forest bird abundance, community structure, and composition following a hurricane in Sweden. Ecography (Cop.), 42, 1–12. 10.1111/ecog.04578

[ece36979-bib-0015] Côté, I. M. , Darling, E. S. , & Brown, C. J. (2016). Interactions among ecosystem stressors and their importance in conservation. Proceedings of the Royal Society B‐Biological Sciences, 283, 1–9. 10.1098/rspb.2015.2592 PMC4760168 26865306

[ece36979-bib-0016] Cross, W. F. , Benstead, J. P. , Rosemond, A. D. , & Bruce Wallace, J. (2003). Consumer‐resource stoichiometry in detritus‐based streams. Ecology Letters, 6, 721–732. 10.1046/j.1461-0248.2003.00481.x

[ece36979-bib-0017] Cross, W. F. , Hood, J. M. , Benstead, J. P. , Huryn, A. D. , & Nelson, D. (2015). Interactions between temperature and nutrients across levels of ecological organization. Global Change Biology, 21, 1025–1040. 10.1111/gcb.12809 25400273

[ece36979-bib-0018] Cummins, K. W. (2016). Combining taxonomy and function in the study of stream macroinvertebrates. Journal of Limnology, 75, 235–241. 10.4081/jlimnol.2016.1373

[ece36979-bib-0019] Davis, J. , Sim, L. , & Chambers, J. (2010). Multiple stressors and regime shifts in shallow aquatic ecosystems in antipodean landscapes. Freshwater Biology, 55, 5–18. 10.1111/j.1365-2427.2009.02376.x

[ece36979-bib-0020] Davis, S. J. , Ó hUallacháin, D. , Mellander, P.‐E. , Kelly, A.‐M. , Matthaei, C. D. , Piggott, J. J. , & Kelly‐Quinn, M. (2018). Multiple‐stressor effects of sediment, phosphorus and nitrogen on stream macroinvertebrate communities. Science of the Total Environment, 637–638, 577–587. 10.1016/j.scitotenv.2018.05.052 29754091

[ece36979-bib-0021] Death, R. G. , Dewson, Z. S. , & James, A. B. W. (2009). Is structure or function a better measure of the effects of water abstraction on ecosystem integrity? Freshwater Biology, 54, 2037–2050. 10.1111/j.1365-2427.2009.02182.x

[ece36979-bib-0022] Ding, N. , Yang, W. , Zhou, Y. , González‐Bergonzoni, I. , Zhang, J. , Chen, K. , Vidal, N. , Jeppesen, E. , Liu, Z. , & Wang, B. (2017). Different responses of functional traits and diversity of stream macroinvertebrates to environmental and spatial factors in the Xishuangbanna watershed of the upper Mekong River Basin, China. Science of the Total Environment, 574, 288–299. 10.1016/j.scitotenv.2016.09.053 27639026

[ece36979-bib-0023] Dolédec, S. , Phillips, N. , & Townsend, C. (2011). Invertebrate community responses to land use at a broad spatial scale: Trait and taxonomic measures compared in New Zealand rivers. Freshwater Biology, 56, 1670–1688. 10.1111/j.1365-2427.2011.02597.x

[ece36979-bib-0024] Dudgeon, D. (2019). Multiple threats imperil freshwater biodiversity in the Anthropocene. Current Biology, 29, R960–R967. 10.1016/j.cub.2019.08.002 31593677

[ece36979-bib-0025] Dudgeon, D. , Arthington, A. H. , Gessner, M. O. , Kawabata, Z.‐I. , Knowler, D. J. , Lévêque, C. , Naiman, R. J. , Prieur‐Richard, A.‐H. , Soto, D. , Stiassny, M. L. J. , & Sullivan, C. A. (2006). Freshwater biodiversity: Importance, threats, status and conservation challenges. Biological Reviews, 81, 163 10.1017/S1464793105006950 16336747

[ece36979-bib-0026] Elbrecht, V. , Beermann, A. J. , Goessler, G. , Neumann, J. , Tollrian, R. , Wagner, R. , Wlecklik, A. , Piggott, J. J. , Matthaei, C. D. , & Leese, F. (2016). Multiple‐stressor effects on stream invertebrates: A mesocosm experiment manipulating nutrients, fine sediment and flow velocity. Freshwater Biology, 61, 362–375. 10.1111/fwb.12713

[ece36979-bib-0027] European Environment Agency . (2015). Freshwater quality — nutrients in rivers. https://www.eea.europa.eu/soer/2015/countries‐comparison/freshwater

[ece36979-bib-0028] Frainer, A. , Jabiol, J. , Gessner, M. O. , Bruder, A. , Chauvet, E. , & McKie, B. G. (2016). Stoichiometric imbalances between detritus and detritivores are related to shifts in ecosystem functioning. Oikos, 125(6), 861–871. 10.1111/oik.02687

[ece36979-bib-0029] Frainer, A. , Polvi, L. E. , Jansson, R. , & McKie, B. G. (2018). Enhanced ecosystem functioning following stream restoration: The roles of habitat heterogeneity and invertebrate species traits. Journal of Applied Ecology, 55, 377–385. 10.1111/1365-2664.12932

[ece36979-bib-0030] Frost, P. C. , & Elser, J. J. (2002). Growth responses of littoral mayflies to the phosphorus content of their food. Ecology Letters, 5, 232–240. 10.1046/j.1461-0248.2002.00307.x

[ece36979-bib-0031] Garson, D. (2015). Multivariate GLM, MANOVA, and MANCOVA 2015 edition. Asheboro, North Carolina, USA: Statistical Associates Publishers.

[ece36979-bib-0032] Gordon, L. J. , Peterson, G. D. , & Bennett, E. M. (2008). Agricultural modifications of hydrological flows create ecological surprises. Trends in Ecology & Evolution, 24, 211–219. 10.1016/j.tree.2007.11.011 18308425

[ece36979-bib-0033] Gratton, C. , & Zanden, M. J. V. (2009). Flux of aquatic insect productivity to land: Comparison of lentic and lotic ecosystems. Ecology, 90, 2689–2699. 10.1890/08-1546.1 19886479

[ece36979-bib-0034] Grimm, N. B. (1988). Role of macroinvertebrates in nitrogen dynamics of a desert stream. Ecology, 69, 1884–1893. 10.2307/1941165

[ece36979-bib-0035] Guzman, L. M. , Germain, R. M. , Forbes, C. , Straus, S. , O’Connor, M. I. , Gravel, D. , Srivastava, D. S. , & Thompson, P. L. (2019). Towards a multi‐trophic extension of metacommunity ecology. Ecology Letters, 22, 19–33. 10.1111/ele.13162 30370702

[ece36979-bib-0036] Harding, H. R. , Gordon, T. A. C. , Eastcott, E. , Simpson, S. D. , & Radford, A. N. (2019). Causes and consequences of intraspecific variation in animal responses to anthropogenic noise. Behavioral Ecology, 30(6), 1501–1511. 10.1093/beheco/arz114 31723315 PMC6838653

[ece36979-bib-0037] Heathwaite, A. L. (2010). Multiple stressors on water availability at global to catchment scales: Understanding human impact on nutrient cycles to protect water quality and water availability in the long term. Freshwater Biology, 55, 241–257. 10.1111/j.1365-2427.2009.02368.x

[ece36979-bib-0038] Hirst, A. G. , Glazier, D. S. , & Atkinson, D. (2014). Body shape shifting during growth permits tests that distinguish between competing geometric theories of metabolic scaling. Ecology Letters, 17, 1274–1281. 10.1111/ele.12334 25060740

[ece36979-bib-0039] Horváth, Z. , Ptacnik, R. , Vad, C. F. , & Chase, J. M. (2019). Habitat loss over six decades accelerates regional and local biodiversity loss via changing landscape connectance. Ecology Letters, 22, 1019–1027. 10.1111/ele.13260 30932319 PMC6518933

[ece36979-bib-0040] Huryn, A. D. , & Benstead, J. P. (2019). Seasonal changes in light availability modify the temperature dependence of secondary production in an Arctic stream. Ecology, 100(6), e02690 10.1002/ecy.2690 30854634

[ece36979-bib-0041] Juvigny‐Khenafou, N. P. D. , Zhang, Y. , Piggott, J. J. , Atkinson, D. , Matthaei, C. D. , Van Bael, S. A. , & Wu, N. (2020). Anthropogenic stressors affect fungal more than bacterial communities in decaying leaf litter: A stream mesocosm experiment. Science of the Total Environment, 716, 135053 10.1016/j.scitotenv.2019.135053 31859062

[ece36979-bib-0042] Kardol, P. , Fanin, N. , & Wardle, D. A. (2018). Long‐term effects of species loss on community properties across contrasting ecosystems. Nature, 557, 710–715. 10.1038/s41586-018-0138-7 29795345

[ece36979-bib-0043] Laliberté, E. , & Legendre, P. (2010). A distance‐based framework for measuring functional diversity from multiple traits. Ecology, 91, 299–305. 10.1890/08-2244.1 20380219

[ece36979-bib-0044] Laliberté, E. , Legendre, P. , & Shipley, B. (2014). FD: Measuring functional diversity from multiple traits, and other tools for functional ecology. R Packag. version 1.0‐12. https://cran.r‐project.org/web/packages/FD/FD.pdf 10.1890/08-2244.1 20380219

[ece36979-bib-0045] Lange, K. , Meier, P. , Trautwein, C. , Schmid, M. , Robinson, C. T. , Weber, C. , & Brodersen, J. (2018). Basin‐scale effects of small hydropower on biodiversity dynamics. Frontiers in Ecology and the Environment, 16, 397–404. 10.1002/fee.1823

[ece36979-bib-0046] Li, Z. , Wang, J. , Liu, Z. , Meng, X. , Heino, J. , Jiang, X. , Xiong, X. , Jiang, X. , & Xie, Z. (2019). Different responses of taxonomic and functional structures of stream macroinvertebrate communities to local stressors and regional factors in a subtropical biodiversity hotspot. Science of the Total Environment, 655, 1288–1300. 10.1016/j.scitotenv.2018.11.222 30577121

[ece36979-bib-0047] Li, Z. , Xing, Y. , Liu, Z. , Chen, X. , Jiang, X. , Xie, Z. , & Heino, J. (2020). Seasonal changes in metacommunity assembly mechanisms of benthic macroinvertebrates in a subtropical river basin. Science of the Total Environment, 729, 139046 10.1016/j.scitotenv.2020.139046 32498180

[ece36979-bib-0048] Lindenmayer, D. B. , Likens, G. E. , Krebs, C. J. , & Hobbs, R. J. (2010). Improved probability of detection of ecological “surprises”. Proceedings of the National Academy of Sciences of the United States of America, 107, 21957–21962. 10.1073/pnas.1015696107 21098660 PMC3009814

[ece36979-bib-0049] Liu, S. , Xie, G. , Wang, L. , Cottenie, K. , Liu, D. , & Wang, B. (2016). Different roles of environmental variables and spatial factors in structuring stream benthic diatom and macroinvertebrate in Yangtze River Delta, China. Ecological Indicators, 61, 602–611. 10.1016/j.ecolind.2015.10.011

[ece36979-bib-0050] Magbanua, F. S. , Townsend, C. R. , Hageman, K. J. , & Matthaei, C. D. (2013). Individual and combined effects of fine sediment and the herbicide glyphosate on benthic macroinvertebrates and stream ecosystem function. Freshwater Biology, 58, 1729–1744. 10.1111/fwb.12163

[ece36979-bib-0051] Matthaei, C. D. , Piggott, J. J. , & Townsend, C. R. (2010). Multiple stressors in agricultural streams: Interactions among sediment addition, nutrient enrichment and water abstraction. Journal of Applied Ecology, 47, 639–649. 10.1111/j.1365-2664.2010.01809.x

[ece36979-bib-0052] MEP . (2002). Environmental quality standard of surface water (GB3838‐2002). http://english.mee.gov.cn/standards_reports/standards/water_environment/quality_standard/200710/t20071024_111792.htm

[ece36979-bib-0053] Merritt R.W., Cummins K.W., & Berg M.B. (Eds.) (2008). An Introduction to the Aquatic Insects of North America, 4th ed Dubuque: Kendall/Hunt.

[ece36979-bib-0054] Mor, J.‐R. , Dolédec, S. , Acuña, V. , Sabater, S. , & Muñoz, I. (2019). Invertebrate community responses to urban wastewater effluent pollution under different hydromorphological conditions. Environmental Pollution, 252, 483–492. 10.1016/j.envpol.2019.05.114 31158676

[ece36979-bib-0055] Nakagawa, S. (2004). A farewell to Bonferroni: The problems of low statistical power and publication bias. Behavioral Ecology, 15, 1044–1045. 10.1093/beheco/arh107

[ece36979-bib-0200] Nakagawa, S. , & Cuthill, I. C. (2007). Effect size, confidence interval and statistical significance: A practical guide for biologists. Biological Reviews, 82, 591–605. 17944619 10.1111/j.1469-185X.2007.00027.x

[ece36979-bib-0056] Nõges, P. , Argillier, C. , Borja, Á. , Garmendia, J. M. , Hanganu, J. , Kodeš, V. , Pletterbauer, F. , Sagouis, A. , & Birk, S. (2016). Quantified biotic and abiotic responses to multiple stress in freshwater, marine and ground waters. Science of the Total Environment, 540, 43–52. 10.1016/j.scitotenv.2015.06.045 26116411

[ece36979-bib-0057] Oksanen, J. , Blanchet, F. G. , Kindt, R. , Legendre, P. , Minchin, P. R. , O'Hara, R. B. , Simpson, G. L. , Solymos, P. , Stevens, M. H. , & Wagner, H. (2015). Vegan: community ecology package. R package version 2.5.6. 1–29. https://cran.r‐project.org/web/packages/vegan/vegan.pdf

[ece36979-bib-0058] Orr, J. A. , Vinebrooke, R. D. , Jackson, M. C. , Kroeker, K. J. , Kordas, R. L. , Mantyka‐Pringle, C. , Van den Brink, P. J. , De Laender, F. , Stoks, R. , Holmstrup, M. , Matthaei, C. D. , Monk, W. A. , Penk, M. R. , Leuzinger, S. , Schäfer, R. B. , & Piggott, J. J. (2020). Towards a unified study of multiple stressors: Divisions and common goals across research disciplines. Proceedings of the Royal Society B‐Biological Sciences, 287, 20200421 10.1098/rspb.2020.0421 PMC7282922 32370677

[ece36979-bib-0059] Ott, D. , Digel, C. , Rall, B. C. , Maraun, M. , Scheu, S. , & Brose, U. (2014). Unifying elemental stoichiometry and metabolic theory in predicting species abundances. Ecology Letters, 17, 1247–1256. 10.1111/ele.12330 25041038

[ece36979-bib-0060] Petsch, D. K. , Schneck, F. , & Melo, A. S. (2017). Substratum simplification reduces beta diversity of stream algal communities. Freshwater Biology, 62, 205–213. 10.1111/fwb.12863

[ece36979-bib-0061] Piggott, J. J. , Salis, R. K. , Lear, G. , Townsend, C. R. , & Matthaei, C. D. (2015). Climate warming and agricultural stressors interact to determine stream periphyton community composition. Global Change Biology, 21, 206–222. 10.1111/gcb.12861 24942814

[ece36979-bib-0062] Piggott, J. J. , Townsend, C. R. , & Matthaei, C. D. (2015). Climate warming and agricultural stressors interact to determine stream macroinvertebrate community dynamics. Global Change Biology, 21, 1887–1906. 10.1111/gcb.12861 25581853

[ece36979-bib-0063] Pillar, V. D. , Blanco, C. C. , Müller, S. C. , Sosinski, E. E. , Joner, F. , & Duarte, L. D. S. (2013). Functional redundancy and stability in plant communities. Journal of Vegetation Science, 24, 963–974. 10.1111/jvs.12047

[ece36979-bib-0064] Poff, N. L. (1997). Landscape filters and species traits: Towards mechanistic understanding and prediction in stream ecology. Journal of the North American Benthological Society, 16, 391–409.

[ece36979-bib-0065] Poff, N. L. , Olden, J. D. , Vieira, N. K. M. , Finn, D. S. , Mark, P. , Kondratieff, B. C. , Poff, N. L. , & Olden, J. D. (2006). Functional trait niches of North American lotic insects: Traits‐based ecological applications in light of phylogenetic relationships. Journal of the North American Benthological Society, 25, 730–755.

[ece36979-bib-0201] Quinn, G. P. , & Keough, M. J. (2002). Experimental design and data analysis for biologists. New York, NY, USA: Cambridge university press.

[ece36979-bib-0066] Rabení, C. F. , Doisy, K. E. , & Zweig, L. D. (2005). Stream invertebrate community functional responses to deposited sediment. Aquatic Sciences, 67, 395–402. 10.1007/s00027-005-0793-2

[ece36979-bib-0067] Salis, R. K. , Bruder, A. , Piggott, J. J. , Summerfield, T. C. , & Matthaei, C. D. (2017). High‐throughput amplicon sequencing and stream benthic bacteria: Identifying the best taxonomic level for multiple‐stressor research. Scientific Reports, 7, 44657 10.1038/srep44657 28327636 PMC5361126

[ece36979-bib-0068] Sato, T. , El‐Sabaawi, R. W. , Campbell, K. , Ohta, T. , & Richardson, J. S. (2016). A test of the effects of timing of a pulsed resource subsidy on stream ecosystems. Journal of Animal Ecology, 85, 1136–1146. 10.1111/1365-2656.12516 26972564

[ece36979-bib-0069] Schäfer, R. B. , Kühn, B. , Hauer, L. , & Kattwinkel, M. (2017). Assessing recovery of stream insects from pesticides using a two‐patch metapopulation model. Science of the Total Environment, 609, 788–798. 10.1016/j.scitotenv.2017.07.222 28768211

[ece36979-bib-0070] Schmidt‐Kloiber, A. , & Hering, D. (2015). www.freshwaterecology.info – An online tool that unifies, standardises and codifies more than 20,000 European freshwater organisms and their ecological preferences. Ecological Indicators, 53, 271–282. 10.1016/j.ecolind.2015.02.007

[ece36979-bib-0071] Statzner, B. , & Bêche, L. A. (2010). Can biological invertebrate traits resolve effects of multiple stressors on running water ecosystems? Freshwater Biology, 55, 80–119. 10.1111/j.1365-2427.2009.02369.x

[ece36979-bib-0072] Sterner, R. W. , Hagemeier, D. D. , Smith, W. L. , & Smith, R. F. (1993). Phytoplankton nutrient limitation and food quality for Daphnia. Limnology and Oceanography, 38, 857–871. 10.1111/j.1600-0587.1999.tb00573.x

[ece36979-bib-0073] Tolonen, K. E. , Leinonen, K. , Marttila, H. , Erkinaro, J. , & Heino, J. (2017). Environmental predictability of taxonomic and functional community composition in high‐latitude streams. Freshwater Biology, 62, 1–16. 10.1111/fwb.12832

[ece36979-bib-0074] Tonkin, J. D. , Heino, J. , & Altermatt, F. (2018). Metacommunities in river networks: The importance of network structure and connectivity on patterns and processes. Freshwater Biology, 63, 1–5. 10.1111/fwb.13045

[ece36979-bib-0075] Townsend, C. R. , & Hildrew, A. G. (1994). Species traits in relation to a habitat templet for river systems. Freshwater Biology, 31, 265–275. 10.1111/j.1365-2427.1994.tb01740.x

[ece36979-bib-0076] Villéger, S. , Mason, N. H. , & Mouillot, D. (2008). New multidimentional functional diversity indices for a multifaceted framework in functional ecology. Ecology, 89, 2290–2301. 18724739 10.1890/07-1206.1

[ece36979-bib-0077] Wagenhoff, A. , Townsend, C. R. , & Matthaei, C. D. (2012). Macroinvertebrate responses along broad stressor gradients of deposited fine sediment and dissolved nutrients: A stream mesocosm experiment. Journal of Applied Ecology, 49, 892–902. 10.1111/j.1365-2664.2012.02162.x

[ece36979-bib-0078] Wilkinson, C. L. , Yeo, D. C. J. , Tan, H. H. , Fikri, A. H. , & Ewers, R. M. (2018). Land‐use change is associated with a significant loss of freshwater fish species and functional richness in Sabah, Malaysia. Biological Conservation, 222, 164–171. 10.1016/j.biocon.2018.04.004

[ece36979-bib-0079] Wood, P. J. , & Armitage, P. D. (1997). Biological effects of fine sediment in the lotic environment. Environmental Management, 21, 203–217. 10.1002/hyp.7604 9008071

[ece36979-bib-0080] Woodward, G. , Gessner, M. O. , Giller, P. S. , Gulis, V. , Hladyz, S. , Lecerf, A. , Malmqvist, B. , McKie, B. G. , Tiegs, S. D. , Cariss, H. , Dobson, M. , Elosegi, A. , Ferreira, V. , Graça, M. A. S. , Fleituch, T. , Lacoursière, J. O. , Nistorescu, M. , Pozo, J. , Risnoveanu, G. , … Chauvet, E. (2012). Continental‐scale effects of nutrient pollution on stream ecosystem functioning. Science (80‐), 336, 1438–1440. 10.1126/science.1219534 22700929

[ece36979-bib-0081] Woodward, G. , Perkins, D. M. , & Brown, L. E. (2010). Climate change and freshwater ecosystems: Impacts across multiple levels of organization. Philosophical Transactions of the Royal Society B: Biological Sciences, 365, 2093–2106. 10.1098/rstb.2010.0055 PMC2880135 20513717

[ece36979-bib-0082] Wu, N. , Thodsen, H. , Andersen, H. E. , Tornbjerg, H. , Baattrup‐Pedersen, A. , & Riis, T. (2019). Flow regimes filter species traits of benthic diatom communities and modify the functional features of lowland streams – a nationwide scale study. Science of the Total Environment, 651, 357–366. 10.1016/j.scitotenv.2018.09.210 30240919

[ece36979-bib-0083] Zhang, Y. , & Malmqvist, B. (1997). Phenotypic plasticity in a suspension‐feeding Insect, Simulium lundstromi (Diptera: Simuliidae), in response to current velocity. Oikos, 78, 503–510.

