# Supplementary material for: Impacts of multiple anthropogenic stressors on stream macroinvertebrate community composition and functional diversity
Source: Ecol Evol. 2020 Dec 16;11(1):133–52. doi: 10.1002/ece3.6979 (PMC7790656; doi:10.1002/ece3.6979)
Supplement: Supplementary file 1 — Appendix S1 [file ECE3-11-133-s001.doc]

**Appendix S1**

Figure S1: Average number of benthic invertebrates in the two flow velocity treatments on the two sampling occasions (Error Bars = +/- SE, *n* = 16).

Figure S2: Average evenness (Pielou’s) of the benthic invertebrate community across the experimental treatments. (Error Bars = +/- SE, *n* = 8 per treatment combination).

**Common invertebrate taxa stressor interactions:**

Numbers of Nemouridae (Fig. S3) and dipteran pupae (Fig. S4) remained stable or increased when sediment alone was added but decreased when flow velocity was reduced as well. For Nemouridae, this interaction overrode the weaker, negative sediment main effect (Table 3). Further, the negative effect of flow velocity reduction on Baetidae was stronger in the absence of fine sediment (Fig S5). Ephemerellidae increased in abundance in nutrient-enriched mesocosms at fast flow but decreased when enrichment was combined with reduced flow velocity (Fig S6). Finally, Chironomidae abundance increased with reduced flow velocity in non-enriched and sediment-free channels, whereas they decreased (or were unaffected) with reduced flow velocity in all other treatment combinations (Fig S7). By contrast, Gordiidae abundance was highest when nutrients were added and flow velocity was reduced in the absence of sediment, but lowest when all three stressors were applied (Fig. S8).

Figure S3: Interactive effects of flow velocity reduction and sediment addition on the abundance of Nemouridae.

Figure S4: Interactive effects of flow velocity reduction and sediment addition on the abundance of Diptera pupae.

Figure S5: Interactive effects of flow velocity reduction and sediment addition on the abundance of Baetidae.

Figure S6: Interactive effects of flow velocity reduction and nutrient enrichment on the abundance of Ephemerillidae.

Figure S7: Average number of Chironomidae in the two flow velocity treatments on the two sampling occasions (Error Bars = +/- SE, *n* = 8).

Figure S9: Average number of Gordiidae in the two flow velocity treatments on the two sampling occasions (Error Bars = +/- SE, *n* = 8).

*Stressor effects on functional diversity and traits*

Interactive stressor effects on trait variables were fairly rare, but interactions between sediment and current velocity reduction as well as between nutrients and velocity reduction were seen in the relative abundances of the two morphological traits and those of shredders, respectively. When flow velocity was reduced, the proportion of streamlined individual generally decreased; however, the slope was smaller in mesocosms with sediment added (Fig. S11). The opposite pattern was seen for non-streamlined organisms (Fig. S12). For shredders, relative abundance decreased when nutrients were added at fast flow but this pattern was reversed at reduced flow velocity (Fig. S13).

Figure S10: Flow velocity main effects across sampling dates on the average relative abundance of shredding invertebrates (Error Bars = +/- SE, n=16).

Figure S11: Interactive effects of flow velocity reduction and sediment addition on the relative abundance of streamlined macroinvertebrates.

Figure S12: Interactive effects of flow velocity reduction and sediment addition on the relative abundance of non-streamlined macroinvertebrates.

Figure S13: Interactive effects of flow velocity reduction and nutrient enrichment on the relative abundance of shredding macroinvertebrates.
